# The virulence–transmission relationship in an obligate killer holds under diverse epidemiological and ecological conditions, but where is the tradeoff?

**DOI:** 10.1002/ece3.3532

**Published:** 2017-11-17

**Authors:** Frida Ben‐Ami

**Affiliations:** ^1^ School of Zoology George S. Wise Faculty of Life Sciences Tel Aviv University Tel Aviv Israel

**Keywords:** *Daphnia magna*, epidemiology, evolution of virulence, multiple infections, *Pasteuria ramosa*, semelparous parasite, tradeoff hypothesis, within‐host competition

## Abstract

Parasite virulence is a leading theme in evolutionary biology. Modeling the course of virulence evolution holds the promise of providing practical insights into the management of infectious diseases and the implementation of vaccination strategies. A key element of virulence modeling is a tradeoff between parasite transmission rate and host lifespan. This assumption is crucial for predicting the level of optimal virulence. Here, I test this assumption using the water flea *Daphnia magna* and its castrating and obligate‐killing bacterium *Pasteuria ramosa*. I found that the virulence–transmission relationship holds under diverse epidemiological and ecological conditions. In particular, parasite genotype, absolute and relative parasite dose, and within‐host competition in multiple infections did not significantly affect the observed trend. Interestingly, the relationship between virulence and parasite transmission in this system is best explained by a model that includes a cubic term. Under this relationship, parasite transmission initially peaks and saturates at an intermediate level of virulence, but then it further increases as virulence decreases, surpassing the previous peak. My findings also highlight the problem of using parasite‐induced host mortality as a “one‐size‐fits‐all” measure of virulence for horizontally transmitted parasites, without considering the onset and duration of parasite transmission as well as other equally virulent effects of parasites (e.g., host castration). Therefore, mathematical models may be required to predict whether these particular characteristics of horizontally transmitted parasites can direct virulence evolution into directions not envisaged by existing models.

## 1 INTRODUCTION

How and why virulence (here defined as parasite‐induced host mortality) evolves is a central theme in evolutionary biology. A key element underlying theoretical and empirical studies of virulence evolution is the tradeoff hypothesis (Alizon, Hurford, Mideo, & van Baalen, 2009; Anderson & May, 1982; Ewald, 1983). This hypothesis assumes that higher parasite transmission rate comes at the cost of a shorter infectious period (killing the host earlier), that is, less time for transmission. Of crucial importance for this model is the shape of the tradeoff function. For example, virulence is expected to evolve to an intermediate level if transmission is a saturating (decelerating) function of virulence. In contrast, if the tradeoff function is accelerating, the optimal level of virulence is maximal. Models on the evolution of virulence typically assume saturating tradeoff functions (Alizon et al., 2009; Anderson & May, 1982; Frank, 1996), but few empirical studies have tested this important assumption (Fraser, Hollingsworth, Chapman, de Wolf, & Hanage, 2007; Jensen, Little, Skorping, & Ebert, 2006; Mackinnon, Gandon, & Read, 2008; de Roode, Yates, & Altizer, 2008). Identifying the shape of the tradeoff function is essential if we are to make predictions on the course of virulence evolution—which is of outmost importance for public health, medicine, and agriculture.

The simplicity of the tradeoff hypothesis has been both its source of appeal and Achilles’ heel (Alizon et al., 2009; Ebert & Bull, 2003). A key assumption at the core of this hypothesis is that virulence and parasite transmission are genetically correlated and linked positively with within‐host exploitation. Thus, virulence–transmission relationships are expected to remain similar across all hosts in the population. Another key assumption of the tradeoff hypothesis is that the parasite is directly and horizontally transmitted throughout the infectious period (Anderson & May, 1982). However, this assumption does not hold for a parasite that exhibits a semelparous life history, releasing all its transmission stages in a single event that usually coincides with host death (i.e., obligate killers, Ebert & Weisser, 1997; Day, 2002). Nonetheless, due to the binary nature of its transmission strategy (all or nothing), an obligate killer risks losing everything if, for example, the host is predated. Therefore, an obligate killer still faces a tradeoff between when to kill its host (virulence) and maximizing within‐host exploitation (which is assumed to be positively linked to parasite transmission).

Several studies have shown the existence of optimal transmission at an intermediate level of virulence in parasites that seek to avoid host death to maintain transmission, for example, HIV in humans (Fraser et al., 2007), myxomatosis in rabbits (Mackinnon et al., 2008), protozoan parasite in monarch butterflies (de Roode et al., 2008), as well as in obligate killers (Bérénos, Schmid‐Hempel, & Wegner, 2009; Jensen et al., 2006; Redman, Wilson, & Cory, 2016). One study has also shown that the virulence–transmission relationship can be affected by host genotype (de Roode & Altizer, 2009). However, no study to date has compared the virulence–transmission relationship across different parasite genotypes while controlling for host genotype. Neither has any study investigated the effects of multiple infections on the shape of the tradeoff function. This is important, because multiple infections are the norm rather than the exception in diverse host–parasite systems, and co‐infections also have implications for human health (Balmer & Tanner, 2011).

Multiple infections introduce another layer of complexity, because within‐host competition among co‐infecting parasite strains may influence the rate at which transmission stages are produced within the host (Kümmerli, Jiricny, Clarke, West, & Griffin, 2009; Pollitt et al., 2011), and thus alter the shape of the tradeoff function. Although the virulence–transmission tradeoff has been incorporated into or emerges from many models of multiple infections (Alizon & van Baalen, 2005; van Baalen & Sabelis, 1995; Nowak & May, 1994), there is no prediction a priori as to how the total transmission rate (sum of the individual transmission rates of each of the co‐infecting strains) should behave as a function of the “overall virulence” of co‐infected hosts (Alizon, de Roode, & Michalakis, 2013). This is an inherent weakness of the tradeoff hypothesis, because the relative infectious dose of co‐infecting strains varies, and as a result, the range of competitive outcomes (i.e., co‐infection scenarios) can be wide.

**Figure 1:**
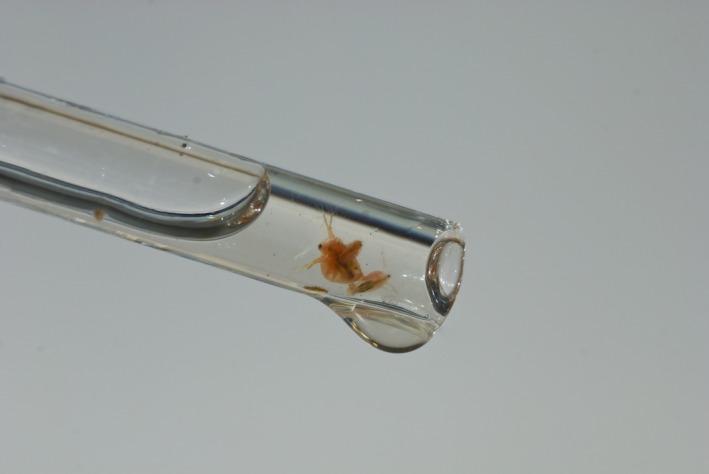
*Daphnia magna* infected with *Pasteuria ramosa*. Photo courtesy of Liron Goren

Here, I analyzed virulence–transmission data from a tractable host–parasite model system, the freshwater planktonic crustacean *Daphnia magna*, and its obligate‐killing bacterial parasite *Pasteuria ramosa* (Figure 1), to study genetic, epidemiological, and ecological aspects potentially influencing the shape of the virulence–transmission relationship. Previous studies of this system have shown that parasite specificity, relative virulence, and relative dose strongly affect the expression and evolution of virulence (Ben‐Ami, Mouton, & Ebert, 2008; Ben‐Ami, Rigaud, & Ebert, 2011; Ben‐Ami & Routtu, 2013). Using a single dose of one *P. ramosa* isolate, an earlier study also found evidence of optimal transmission at an intermediate level of virulence (Jensen et al., 2006). I present a comprehensive analysis of the shape of the tradeoff function under diverse scenarios: (1) infections by single parasite genotypes (clones), (2) infections by parasite isolates (parasite samples from infected hosts that may contain multiple genotypes), and (3) mixed infections. All scenarios are closely tied to the parasite's epidemiology, by including various dose levels in single infections as well as relative doses in multiple infections.

## 2 MATERIALS AND METHODS

### 2.1 Experiment

**Figure 2:**
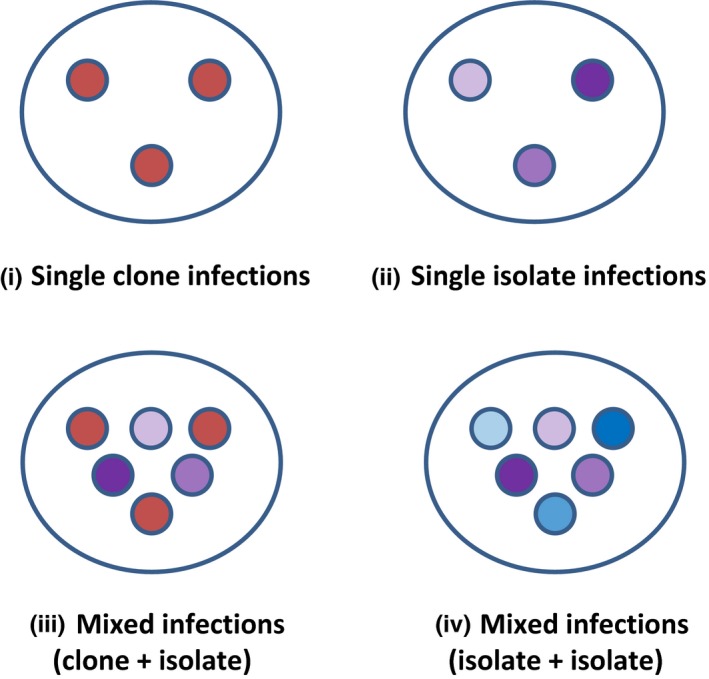
Schematic of the four types of infection treatments analyzed here (Ben‐Ami & Routtu, 2013). The two *P. ramosa* clones C1 and C14 originated from Russia and Finland, respectively. The three *P. ramosa* isolates P1, P2, and P4 originated from Germany, England, and Belgium, respectively. Isolates are parasite samples from infected hosts that may contain multiple genotypes (Luijckx et al., 2011). It is likely that parasite genotypes isolated from infected hosts from one location are more genetically related to each other than to another *P. ramosa* clone or isolate from a geographically distant location. This is why parasite genotypes belonging to the same isolate were marked with different shades of the same color

The experimental setup and design on which this analysis is based are described in detail in Ben‐Ami and Routtu (2013). The effects of mixed infections on virulence and parasite transmission were analyzed in Ben‐Ami and Routtu (2013). Here, I use the data to test the key assumption of the tradeoff hypothesis. In brief, individual *D. magna* were exposed to one of four types of infection treatments (Figure 2): (1) single infections by two *P. ramosa* clones (single genotype) in four dose levels, (2) single infections by three *P. ramosa* isolates (possibly containing multiple genotypes) in the same dose levels, (3) mixed infections by four mixtures each containing a *P. ramosa* clone and a *P. ramosa* isolate in equal (50:50) and unequal proportions (90:10 and 10:90), and (4) mixed infections by two mixtures each containing two *P. ramosa* isolates in the same equal and unequal proportions. Hosts were observed daily for release of offspring and signs of infection, the latter of which is evident by a reddish‐brownish coloration 2 weeks postexposure. I recorded time‐to‐host‐death‐since‐exposure as a measure of parasite‐induced host mortality (virulence). I also quantified the lifetime spore production of an infection (parasite fitness), by crushing dead *Daphnia* and counting the transmission stages (=spores) using a hemocytometer (Thoma chamber).

### 2.2 Quantitative differences among parasite clones and isolates

The here used *P. ramosa* isolates were obtained from infected *Daphnia* collected in Germany (P1), England (P2), and Belgium (P4) (Ben‐Ami & Routtu, 2013). These isolates are known to contain multiple genotypes, as microsatellite analysis revealed different alleles at the same locus within an isolate (Mouton, Nong, Preston, & Ebert, 2007). Additionally, isolates selected for their differences in infectivity reveal few but clear‐cut differences in collagen‐like protein patterns, which often play an important role in attachment to host cells prior to infection (Mouton, Traunecker, McElroy, du Pasquier, & Ebert, 2009). The *P. ramosa* clones C1 and C14 were obtained, respectively, from isolates P5 (Russia) and P3 (Finland) via infection by limited dilution (technical details in Luijckx, Ben‐Ami, Mouton, du Pasquier, and Ebert (2011)). Clones are a single genotype, whereas isolates are parasite samples from infected hosts that may contain multiple genotypes. Given that isolates P1, P3, and P4 did not originate from the same geographical areas as isolates P5 and P3 (from which clones C1 and C4 were obtained, respectively), it is unlikely that the isolates and clones are related. Isolates are a naturally occurring feature of the *Daphnia*–*Pasteuria* host–parasite system and are thus relevant to evolutionary processes in natural populations. Both clones and isolates were propagated through the experimental host clone HO2, to obtain enough spore‐carrying cadavers to produce sufficient amounts of spore suspensions for the experiment. Previously developed primers allow differentiating between a *P. ramosa* clone and a *P. ramosa* isolate and between two *P. ramosa* isolates in the mixed infections treatments (Mouton et al., 2007). Besides these genetic differences, there were also considerable differences in their virulence and lifetime spore production (Ben‐Ami & Routtu, 2013; Ben‐Ami, Mouton, et al. 2008).

### 2.3 Estimating virulence and parasite transmission rate

The tradeoff hypothesis links virulence with parasite transmission rate. Host longevity is a good proxy for parasite‐induced host mortality (virulence), because background (natural) mortality was practically inexistent or very low in the unexposed control groups (Ben‐Ami & Routtu, 2013), and host longevity among exposed individuals that did not acquire infection did not differ from that of controls (Izhar & Ben‐Ami, 2015). To estimate parasite transmission rate, several measures are commonly used in empirical studies: probability of infection (de Roode et al., 2008), percentage of infected hosts (Doumayrou, Avellan, Froissart, & Michalakis, 2013), and parasite load (Chapuis, Arnal, & Ferdy, 2012; Fraser et al., 2007). I first estimated the density‐dependent transmission rate (β) for each parasite clone/isolate using infection data (see Appendix S1). I did not find statistically significant differences in the transmission rate between the two *P. ramosa* clones and among the three *P. ramosa* isolates (Appendix S1: Table S1). However, the transmission rate of *P. ramosa* isolates was higher than that of clones. I then used parasite spore production as an estimate of parasite transmission rate, because β and parasite spore production correlate positively in the *Daphnia*–*Pasteuria* system (Izhar & Ben‐Ami, 2015). Moreover, earlier studies showed that parasite dose affects the probability of infection in this system (Ben‐Ami, Ebert, & Regoes, 2010; Ben‐Ami, Regoes, & Ebert, 2008). Although a single *P. ramosa* spore can cause disease, the likelihood of such an event is extremely low (ca. 1 in 700) (Luijckx et al., 2011). A parasite that produces more transmission stages will have a greater representation in subsequent generations and is thus more likely to transmit.

### 2.4 Statistical analysis

All statistics were performed using R, version 3.4.1 (R Core Team, www.R-project.org). Initially, I checked for overdispersion by looking at the ratio of the sum of squared Pearson residuals over residual degrees of freedom. I then used generalized linear models with a quasi‐Poisson error family and a log link (glm function) to regress parasite spore production on time‐to‐host‐death‐since‐exposure based on the lowest residual deviance. Host offspring counts were entered as a continuous predictor. In single infections, the dichotomous variable parasite clone/isolate was treated as a fixed factor (categorical predictor). It was then entered into regression models to explain possible differences in the relationship between virulence and spore production of *P. ramosa* clones versus isolates. Also in single infections, dose level was treated as a continuous predictor. Nested models (e.g., quadratic vs. linear, cubic vs. quadratic; details further below) were compared using an analysis of deviance (ANOVA function with test = “Chisq”).

The shape of the predicted relationship between time‐to‐host‐death‐since‐exposure and parasite spore production depends on the coefficient estimates of the polynomial terms used for model building. For example, a positive linear term would imply that the optimal level of virulence is minimal, whereas a negative linear term would imply that the optimal level of virulence is maximal. Adding a negative quadratic term would indicate a saturating relationship that is suggestive of optimal transmission at an intermediate level of virulence. Further adding a negative cubic term would still “look” very quadratic, but if the sign of the cubic term is positive and that of the quadratic term is negative, a local optimal transmission would emerge, followed by a further increase in transmission coupled with reduced virulence. This second increase in parasite transmission was found to be significant in most cases (see Appendix S1 and Section 3 below).

## 3 RESULTS

**Figure 3:**
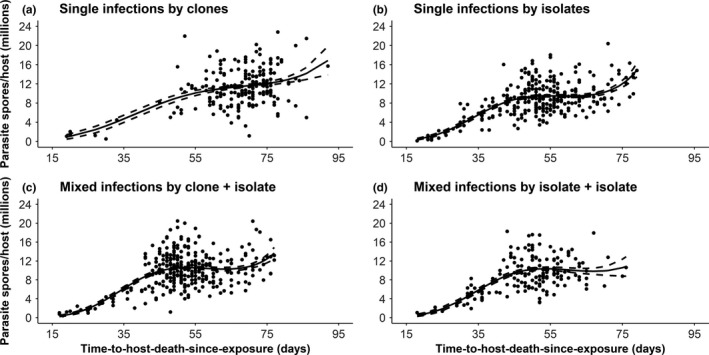
Relationship between time‐to‐host‐death (virulence) and lifetime spore production of an infection (parasite transmission) in (a) single infections by *P. ramosa* clones (b) single infections by *P. ramosa* isolates (c) mixed infections by a clone and an isolate, and (d) mixed infections by two isolates. The solid curve represents predicted values from a generalized linear model with a cubic term that provides the best fit. The dashed curves represent 95% confidence interval for each of the fitted model curves

Nine hundred and thirty‐eight infected *D. magna* were used in the analysis. I found that a generalized linear model with a third‐degree polynomial (cubic term) explains significantly more of the deviance in the data compared to models with first‐ or second‐degree polynomials (linear or quadratic terms; Table 1, Figure 3). Adding a fourth‐degree polynomial term did not improve model fit. This pattern was consistent in all four types of infection treatments. Although the fitted coefficient for host offspring production was always statistically significant (*p *<* *.01), its overall effect size was marginal (i.e., approximately −0.01 in all models). Furthermore, correcting for host fecundity did not cause any of the statistically significant polynomial terms of host longevity to change sign or become nonsignificant.

**Table 1:** Analysis of model deviance with polynomial degrees of first, second, third, and fourth orders for the four types of infection treatments

Degrees	Infection treatments											
	Single infections by clones			Single infections by isolates			Mixed infections by clone + isolate			Mixed infections by isolate + isolate		
	*df*	Deviance	*p*	*df*	Deviance	*p*	*df*	Deviance	*p*	*df*	Deviance	*p*
2 versus 1	196	9.93	**.003**	263	29.42	**<.0001**	304	82.27	**<.0001**	154	42.02	**<.0001**
3 versus 2	195	15.32	**.0003**	262	33.73	**<.0001**	303	27.77	**<.0001**	153	6.46	**.01**
4 versus 3	194	0.02	.895	261	1.53	.181	302	1.18	.261	152	0.03	.865

The null model has a first‐degree polynomial term that is highly significant (*p *<* *.0001) in all infection treatments. Bold typeface indicates significant effects.

**Figure 4:**
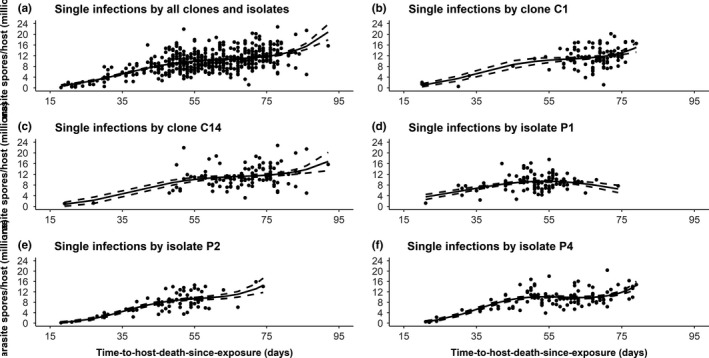
Relationship between time‐to‐host‐death (virulence) and lifetime spore production of an infection (parasite transmission) in single infections by *P. ramosa* clones and isolates: (a) pooling of all clones and isolates, (b) clone C1, (c) clone C14, (d) isolate P1, (e) isolate P2, and (f) isolate P4. The solid curve represents predicted values from a generalized linear model with a cubic term (or quadratic term for isolate P1) that provides the best fit. The dashed curves represent 95% confidence interval for each of the fitted model curves

In the case of infections by a single *P. ramosa* clone or by a single *P. ramosa* isolate, I also entered the parasite clone/isolate and the dose level into the respective models. However, their fitted coefficients were nonsignificant (Table 2). Even though the fitted coefficients for parasite clone and parasite isolate were statistically not significant, I performed regression analyses separately for each parasite clone/isolate. In four of the five cases, a generalized linear model with a cubic term provided the best fit (Table 3, Figure 4). However, for parasite isolate P1 a quadratic term was sufficient to explain the deviance in the data.

**Table 2:** Coefficient estimates, their *t* statistic, and significance level for the model with a cubic term that best explains the deviance in parasite spore production

Coefficient	Infection treatments											
	Single infections by clones			Single infections by isolates			Mixed infections by clone + isolate			Mixed infections by isolate + isolate		
	Estimate	*t*	*p*	Estimate	*t*	*p*	Estimate	*t*	*p*	Estimate	*t*	*p*
Linear	4.39	6.79	**<.0001**	5.57	11.05	**<.0001**	6.27	10.56	**<.0001**	4.44	8.55	**<.0001**
Quadratic	−2.15	−3.27	**.001**	−3.85	−6.96	**<.0001**	−5.63	−8.27	**<.0001**	−3.27	−6.01	**<.0001**
Cubic	1.60	3.57	**.0005**	2.51	6.00	**<.0001**	2.35	5.14	**<.0001**	1.23	2.50	**.01**
Host offspring	−0.015	−2.95	**.004**	−0.012	−4.51	**<.0001**	−0.018	−5.33	**<.0001**	−0.010	−2.38	**.02**
Parasite (1)	0.021	0.44	.659	−0.073	−1.43	.155						
Parasite (2)				−0.016	−0.33	.740						
Dose	0.001	1.42	.159	−0.001	−0.49	.628						

Bold typeface indicates significant coefficient estimates.

**Table 3:** Analysis of model deviance with increasing polynomial degrees for single infection treatments using *P. ramosa* clones and isolates

Degrees	Single infection treatments														
	Parasite clone C1			Parasite clone C14			Parasite isolate P1			Parasite isolate P2			Parasite isolate P4		
	*df*	Deviance	*p*	*df*	Deviance	*p*	*df*	Deviance	*p*	*df*	Deviance	*p*	*df*	Deviance	*p*
**2** versus **1**	96	7.84	**.005**	98	6.00	**.037**	96	8.16	**.003**	62	10.06	**.0008**	100	22.27	**<.0001**
**3** versus **2**	95	4.04	**.044**	97	6.35	**.032**	95	2.08	.128	61	5.74	**.011**	99	22.38	**<.0001**

The null model has a first‐degree polynomial term that is highly significant (*p *<* *.0001) in all infection treatments. Bold typeface indicates significant effects.

## 4 DISCUSSION

I found that the virulence–transmission relationship in the *Daphnia*–*Pasteuria* system holds under diverse epidemiological and ecological conditions. In particular, parasite genotype (clones), epidemiology (absolute and relative dose), and ecology (within‐host competition) did not significantly affect the observed trend. Interestingly, the relationship between virulence and parasite transmission in this system is best explained by a model with a cubic term. Consistent with previous studies, early killing results in the production of few or no transmission stages (Jensen et al., 2006; de Roode et al., 2008). Parasite transmission initially appears to peak at an intermediate level of virulence, after which it saturates or slightly decreases as predicted from optimality models. Thereafter, parasite transmission increases again as virulence decreases, and passes the previous peak. This latter increase would lead to the evolution of lower levels of virulence, because late‐killing parasites that produce more transmission stages will have a greater representation in subsequent generations. This latter increase is particularly beneficial for an obligate killer like *P. ramosa*, whose infectious period begins upon host death.

### 4.1 The generality of the virulence–transmission relationship

My results demonstrate that the virulence–transmission relationship holds under diverse conditions. Under all infection scenarios, transmission stage production increased with time since infection, but also showed the expected deceleration. The shape of the tradeoff function did not differ significantly among parasite clones and isolates. However, the two parasite clones achieved maximal transmission at lower levels of virulence than the three parasite isolates (ca. 65–70 vs. 50–55 days; Figure 4). Under all infection scenarios, host offspring production was negatively correlated with parasite spore production (Table 2). More precisely, the regression models I constructed predict that producing an additional host offspring entails a reduction of about 10,000–18,000 spores. Given that *D. magna* can produce a clutch of up to 100 eggs every 3–4 days, depending on feeding conditions (Ebert, 2005; McKee, 1997), these correlations emphasize the importance for the parasite of efficient (=total) host castration (Jaenike, 1996; O'Keefe & Antonovics, 2002).

Within‐host competition also did not significantly affect the shape of the tradeoff function. The level of virulence evolving results from the interplay between within‐ and between‐host competitions. Distinguishing the causes of virulence evolution, by examining associations among virulence, parasite transmission, and levels of within‐host competition (i.e., multiple infections), has proven to be challenging (Alizon & Michalakis, 2011; Smith, 2011). In this study, mixed infections achieved maximal transmission at levels of virulence similar to that of parasite isolates (ca. 50–55 days; Figure 3), most likely because the more virulent parasite isolates were better competitors than the less virulent clones during mixed infections containing a clone and an isolate (Ben‐Ami & Routtu, 2013). It has been suggested that successful competitors might be able to facultatively upregulate their replication rates upon detection of another genotype within the same host, and thus express higher virulence (Kümmerli et al., 2009; Pollitt et al., 2011). Facultative upregulation of replication rates would be especially beneficial for the parasite clone that is present in a low starting concentration upon infection, although it remains to be determined whether such mechanism is employed by *P. ramosa*. Nevertheless, this study indicates that although the expressed levels of virulence and transmission potential can be affected by within‐host competition, the virulence–transmission relationship observed in single infections may hold under multiple infections. Thus, when making predictions on virulence evolution under conditions of frequent multiple infections, mathematical models may include the same relationship between virulence and parasite transmission that was assumed in single infections.

The consistent shape of the curve may also have a mechanistic explanation. The host can viewed as an ecosystem with a carrying capacity, where the bacterium grows logistically through time (Ebert & Weisser, 1997). The carrying capacity of the host can change if the host exhibits gigantism, because bigger hosts can store more spores and have higher food intake (Baudoin, 1975; Ebert, Carius, Little, & Decaestecker, 2004). Although the size of the *Daphnia* hosts at the time of death is unavailable, the use of a genetically identical host clone in this study limits variations in size at death to phenotypic heterogeneity. This kind of heterogeneity can be caused by internal factors, such as molecular differences in immune response (Brites et al., 2008) and within‐clone variation in life‐history traits (e.g., difference in size at birth), or by external factors (e.g., micro‐environmental variation among the experimental jars).

### 4.2 The biological meaning of a cubic term

While most hosts died around the time when parasite spore production peaked for the first time, some hosts lived much longer up to the point where spore production peaked for the second time. This second peak was usually higher than the first peak, thereby suggesting that late killing of the host could benefit the parasite and constitute an optimal strategy for the parasite. If spore production is highest at very high host ages, why did the majority of the hosts die during the first peak? First, prolonging host lifespan to achieve higher transmission bears the risk of increasing parasite‐independent mortality rates, for example, predation. Put differently, the parasite risks losing “everything” if the host is predated. However, some predators of *Daphnia* (e.g., *Chaoborus* spp. feeding on *Daphnia dentifera*) can disperse spores while feeding, thereby spreading the disease (Cáceres, Knight, & Hall, 2009), whereas other predators can avoid infected *Daphnia* altogether (e.g., *Anisops* sp. feeding on *D. magna*; Goren & Ben‐Ami, 2017). Furthermore, although the survivability of *P. ramosa* spores in the guts of predators of *Daphnia* is unknown, *Pasteuria* spores passed through the gut of *D. magna* remain viable for a “second chance” at infecting a new host (King, Auld, Wilson, James, & Little, 2013). Second, infected *D. magna* may occasionally regain fecundity and produce an additional clutch shortly before death (Magerøy, Grepperud, & Jensen, 2011; L. Goren et al. unpublished data). My results indicate that such increased fecundity bears a cost in the form of reduced spore production. Even if late killing is adaptive for the parasite, because the second spore production peak is higher than the first one, and at the same time allows the host to regain fecundity, selection is likely to be weak because fitness related traits are under weaker selection in older hosts (Hamilton, 1966; Partridge & Barton, 1993; Williams, 1957).

In an earlier test of the virulence–transmission tradeoff in the same host–parasite system, Jensen et al. (2006) showed that a generalized linear model with a quadratic term provides the best fit. They used one host clone–parasite isolate combination under similar conditions as in the present study (39 individuals from *D. magna* clone EL‐75‐69 were exposed to 20,000 parasite spores of the here used isolate P1). Thus, my results for parasite isolate P1 are consistent with Jensen's study. However, there seem to be differences between the results obtained with P1 versus the other *P. ramosa* clones and isolates used in this study with regard to the optimal time to kill the host. These results emphasize the need to use a wider range of host and parasite genotypes, to better understand how G × G interactions can shape the tradeoff function.

### 4.3 The tradeoff hypothesis and obligate killers

The tradeoff hypothesis assumes that the parasite is directly and horizontally transmitted (HT). Parasite‐induced host mortality is the most commonly used measure of virulence for HT parasites (Alizon et al., 2009). By shortening or prolonging the host's lifespan, a HT parasite essentially modulates the duration of infectiousness that is traded off with the rate of parasite transmission. However, the onset of infectiousness among HT parasites varies considerably. Some HT parasites are directly transmitted when the host is alive (from infection to host death or for shorter periods), while others can only be transmitted upon host death or even remain transmissible for many years afterward, for example, via long‐ and free‐living stages (propagules) such as parasite spores buried in pond or lake sediments (Poulin, 2006; Schmid‐Hempel, 2011). This latter transmission method is used by many groups of organisms, including bacteriophages, viruses, bacteria, microsporidia, nematodes, and fungi. If the infectious period can span many years after the host had died, like in the case of *P. ramosa*, then parasite genotypes that produce more transmission stages by prolonging the host's lifespan would be selected over parasite genotypes that produce spores at an optimal rate, provided parasite‐independent mortality rates are such that hosts do not die early before the production of parasite transmission stages had been maximized. In other words, the contribution of shortening the host's lifespan to the overall infectious period would be marginal, and thus intermediate optimal virulence would not necessarily evolve. Instead, the optimal virulence is minimal virulence. The here observed cubic term in the relationship between virulence and parasite transmission seems to support this prediction.

The inclusion of a free‐living stage in the parasite's life cycle can decouple time scales of within‐host reproduction and between‐host transmission, complicating host–parasite dynamics (Caraco & Wang, 2008). The idea that higher parasite propagule survival selects for higher virulence has received considerable theoretical attention (Bonhoeffer, Lenski, & Ebert, 1996; Ewald, 1994; Gandon, 1998; Kamo & Boots, 2004). It has been suggested that if virulence evolves independently of transmission or if multiple infections occur, then long‐lived infective stages can select for higher virulence. But if a tradeoff occurs between virulence and transmission, then no link is predicted (Gandon, 1998; Kamo & Boots, 2004). Based on these predictions, the observed relationship between virulence and transmission in this study would imply that long‐lived *P. ramosa* infective stages need not necessarily select for higher virulence. Yet virulent effects of castrating parasites like *P. ramosa* are among the most fitness‐devastating for the host (Baudoin, 1975; Ebert et al., 2004; O'Keefe & Antonovics, 2002; Obrebski, 1975). One possible explanation for this apparent contradiction may be the definition that is being used for virulence, because the effects of host castration on host fitness are as severe as those of parasite‐induced host mortality. It remains to be determined if virulence (defined as host castration) evolves independently of parasite transmission as predicted by theory. Alternatively, the higher virulence of *P. ramosa* may be due to the commonality of multiple infections in natural populations (Andras & Ebert, 2013; Goren & Ben‐Ami, 2013; Mouton et al., 2007), which is in line with theoretical predictions.

## 5 CONCLUSIONS

This study shows that virulence–transmission relationships can remain consistent across different parasite genotypes and under diverse epidemiological conditions. This study also indicates that multiple infections do not necessarily affect these relationships. However, for a tradeoff to emerge it is important to consider the timing and duration of parasite transmission (e.g., parasites with a semelparous life‐history or long‐lived infective stages). Caution should be exercised when using parasite‐induced host mortality as a “one‐size‐fits‐all” measure of virulence for HT parasites, without considering equally virulent effects of parasites (e.g., host castration). Therefore, mathematical models may be required to predict whether these particular characteristics of HT parasites can direct virulence evolution into directions not envisaged by existing models.

## CONFLICT OF INTEREST

None declared.

## AUTHOR CONTRIBUTIONS

FBA conceived and designed the study, performed data and statistical analyses, and wrote the manuscript.
