# Supplementary material for: The virulence–transmission relationship in an obligate killer holds under diverse epidemiological and ecological conditions, but where is the tradeoff?
Source: Ecol Evol. 2017 Nov 17;7(24):11157–66. doi: 10.1002/ece3.3532 (PMC5743645; doi:10.1002/ece3.3532)
Supplement: Supplementary file 1 [file ECE3-7-11157-s001.docx]

SUPPORTING INFORMATION

for

**The virulence-transmission relationship in an obligate killer holds under diverse epidemiological and ecological conditions, but where is the trade-off?**

Frida Ben-Ami

School of Zoology, George S. Wise Faculty of Life Sciences, Tel Aviv University, Tel Aviv 6997801, Israel

Tel: +972-3-6406080, Fax: +972-3-6405347, e-mail: [frida@post.tau.ac.il](mailto:frida@post.tau.ac.il)

**Estimation of the density-dependent transmission rate, *β***

I estimated the density-dependent transmission rate (*β*) for each parasite clone/isolate using my infection data. To this end, I fit a simplified ODE model which assumed no parasite spore loss and no births or deaths of hosts ([Civitello et al., 2012](#_ENREF_3), [Fenton et al., 2002](#_ENREF_6), [Hall et al., 2007](#_ENREF_7)):

$$\frac{dS}{dt}=-\beta SZ \left( 1 \right)$$

$$\frac{dI}{dt}=-\beta SZ (2)$$

where *β* is the transmission rate parameter, and *S*, *I* and *Z* denote densities of susceptible hosts, infected hosts and parasite spores, respectively. I ignored the death rate of the parasite during exposure, since *P. ramosa* spores can survive several decades ([Decaestecker et al., 2004](#_ENREF_5)). Analytically solving the ODE for susceptible hosts provides a prediction for *S(t)*, the number of susceptible hosts at time *t* (=exposure duration), given *β*, the initial density of susceptible hosts, *S(0)*, and the initial density of parasite spores, *Z(0)* ([Civitello et al., 2012](#_ENREF_3), [Civitello et al., 2013](#_ENREF_4)):

$$S\left( t \right)=S\left( 0 \right)e^{-\beta Z\left( 0 \right)t} (3)$$

In this study, I infected *Daphnia* using four dose levels (10,000, 50,000, 90,000 and 100,000 spores per animal in 20 mL of medium) from every parasite clone/isolate. The duration of exposure was seven days. The probability of remaining uninfected, *p*, is thus determined by *β*:

$$p=\frac{S\left( 7 \right)}{S\left( 0 \right)}=e^{-\beta\cdot Z\left( 0 \right)\cdot7} (4)$$

To find the maximum likelihood estimates for *β* for every parasite clone/isolate, I summed the negative log-likelihood calculated for the four dose levels. Then, for each parasite clone/isolate I determined the value of *β* that minimized this total using the mle2 function of the bbmle package in R (Table S1) ([Bolker, 2008](#_ENREF_1), [Bolker, 2017](#_ENREF_2)). I also calculated 95% confidence intervals for all *β* using the profile and confint functions of the bbmle package (Table S1) ([Bolker, 2008](#_ENREF_1), [Bolker, 2017](#_ENREF_2)).

**Table S1.** Maximum likelihood estimates (MLE) of the density-dependent transmission rate, *β*, for two *P. ramosa* clones (C1, C14) and three *P. ramosa* isolates (P1, P2, P4). CI=confidence interval. NLL=negative log-likelihood of the models fit to the respective datasets.

| **Parasite clone/isolate** | ***β* (spore^-1^day^-1^mL)** | **95% CI** | **NLL** |
| --- | --- | --- | --- |
| C1 | 0.00023 | 0.00016, 0.00033 | -10.42 |
| C14 | 0.00023 | 0.00022, 0.00024 | -10.42 |
| P1 | 0.00094 | 0.00053, 0.00174 | -0.98 |
| P2 | 0.00095 | 0.00055, 0.00177 | -0.98 |
| P4 | 0.00058 | 0.00034, 0.00088 | -1.58 |

**Relationship between time-to-host-death-since-exposure and parasite spore production from day 55 onwards**

Here I analyzed the data corresponding to a particular window of time-to-host-death-since-exposure, which corresponds to the second increase in parasite spore production, to test if this second increase was significant at very low virulence. I used day 55 as the beginning of the window, as this day was shown previously to be the optimal time to kill the host in this system ([Jensen et al., 2006](#_ENREF_8)). This day also corresponds to the flattening of the curve following the first increase in parasite spore production. I found the relationship during the second increase to be significant in single infections by clones (t=2.892, df=180, *P*=0.0043; Figure 3A) and single infections by isolates (t=2.702, df=111, *P*=0.00798; Figure 3B), as well as marginally significant in mixed infections by clone+isolate (t=1.856, df=100, *P*=0.0664; Figure 3C). Only in mixed infections by isolate+isolate, the relationship was found to be non-significant (t=-0.436, df=41, *P*=0.665; Figure 3D), probably because the relationship for isolate P1 was quadratic (Table 3, Figure 4D).

**References**

BOLKER, B. M. 2008. *Ecological models and data in R,* Princeton, NJ, Princeton University Press.

BOLKER, B. M. 2017. bbmle: Tools for general maximum likelihood estimation. <http://cran.r-project.org/web/packages/bbmle/index.html>.

CIVITELLO, D. J., FORYS, P., JOHNSON, A. P. & HALL, S. R. 2012. Chronic contamination decreases disease spread: a *Daphnia-*fungus-copper case study. *Proc. R. Soc. B.,* 279**,** 3146-3153.

CIVITELLO, D. J., PENCZYKOWSKI, R. M., HITE, J. L., DUFFY, M. A. & HALL, S. R. 2013. Potassium stimulates fungal epidemics in a freshwater invertebrate. *Ecology,* 94**,** 380-388.

DECAESTECKER, E., LEFEVER, C., DE MEESTER, L. & EBERT, D. 2004. Haunted by the past: evidence for resting stage banks of microparasites and epibionts of *Daphnia*. *Limnol. Oceanogr.,* 49**,** 1355-1364.

FENTON, A., FAIRBAIRN, J. P., NORMAN, R. & HUDSON, P. J. 2002. Parasite transmission: reconciling theory and reality. *J. Anim. Ecol.,* 71**,** 893-905.

HALL, S. R., SIVARS-BECKER, L., BECKER, C., DUFFY, M. A., TESSIER, A. J. & CÁCERES, C. E. 2007. Eating yourself sick: transmission of disease as a function of foraging ecology. *Ecol. Lett.,* 10**,** 207-218.

JENSEN, K. H., LITTLE, T. J., SKORPING, A. & EBERT, D. 2006. Empirical support for optimal virulence in a castrating parasite. *PLoS Biol.,* 4**,** 1265-1269.
